# The Effect of Monobenzone Cream on Oxidative Stress and Its Relationship With Serum Levels of IL‐1β and IL‐18 in Vitiligo Patients

**DOI:** 10.1111/jocd.16544

**Published:** 2024-09-23

**Authors:** Ahmed Khalid‐Meften, Mahsa Liaghat, Mohammad Yazdanpour, Mohsen Nabi‐Afjadi, Asieh Hosseini, Elham Bahreini

**Affiliations:** ^1^ Department of Biochemistry, Faculty of Medicine Iran University of Medical Sciences Tehran Iran; ^2^ Department of Medical Laboratory Sciences, Faculty of Medical Sciences, Kazerun Branch Islamic Azad University Kazerun Iran; ^3^ Department of Molecular Genetics, Faculty of Biological Sciences Tarbiat Modares University Tehran Iran; ^4^ Department of Biochemistry, Faculty of Biological Sciences Tarbiat Modares University Tehran Iran; ^5^ Razi Drug Research Center Iran University of Medical Sciences Tehran Iran

**Keywords:** inflammation, malondialdehyde, monobenzyl ether of hydroquinone, oxidative stress, total oxidant status, vitiligo

## Abstract

**Background:**

Monobenzyl ether hydroquinone (MEBHQ) is a cream that promotes the spread and evenness of skin patches in vitiligo. Our aim was to investigate the oxidative and inflammatory effects of this cream on vitiligo patients consuming MEBHQ.

**Methods:**

A case–control study was conducted with three groups of 30 people from the control group, vitiligo patients before and after treatment. The percentage of vitiligo spots was determined by a specialist doctor. The levels of biochemical factors, oxidative stress profile and inflammatory factors were measured by enzymatic, colorimetric and ELISA methods, respectively.

**Results:**

Vitiligo patients showed a high level of inflammation and oxidative stress compared to healthy people. Although after 3 months of using MBEHQ cream, the percentage of skin spots in vitiligo patients increased from an average of 63%–91% and the skin color became almost uniform, but it still increased the level of oxidative stress and inflammation in these patients. Although the level of oxidative stress increased significantly in these patients, there was no significant increase in the level of malondialdehyde. The lack of significant differences in the levels of biochemical factors between healthy people and vitiligo patients before and after using the treatment shows the absence of side effects.

**Conclusion:**

The use of MBEHQ increased the size of skin spots and uneven skin color in vitiligo patients. Although MBEHQ did not show side effects such as diabetes, liver and kidney diseases, it increased the levels of oxidative stress and inflammatory cytokines, which needs further study.

## 1 Introduction

Vitiligo is a long‐term skin condition characterized by patches of the skin losing their pigment. It occurs when melanocytes, the cells responsible for producing melanin, are destroyed or stop functioning. This results in white or depigmented patches on the skin. Vitiligo can affect any part of the body and can vary in size and severity. The exact cause of vitiligo is not fully understood, but it is believed to be an autoimmune condition where the body's immune system mistakenly attacks and destroys melanocytes [1].

There are different types of vitiligo, which are classified based on the distribution and pattern of depigmented patches on the skin [2]. The main types of vitiligo include the following. (1) Non‐segmental vitiligo, the most common form of vitiligo, is characterized by depigmented patches that appear on both sides of the body in a symmetrical pattern. These patches can occur on the face, hands, arms, feet, and other parts of the body. (2) Segmental vitiligo, which is characterized by depigmented patches that are localized to one side or segment of the body. It typically affects younger individuals and may progress rapidly before stabilizing. (3) Mixed vitiligo, in which features of both non‐segmental and segmental vitiligo are combined, with the depigmented patches appearing in a mixed pattern on the body. (4) Universal vitiligo, a rare and severe form of vitiligo in which depigmented patches cover most of the body, resulting in extensive loss of skin color [3].

The vitiligo therapy can involve both depigmentation and repigmentation approaches, depending on the individual's specific needs and preferences [1]. Depigmentation therapy, such as the use of monobenzyl ether hydroquinone (MBEHQ), is typically considered in cases of extensive vitiligo where depigmenting the remaining normal skin can help achieve a more uniform skin tone. This approach aims to lighten the unaffected areas of the skin to match the depigmented patches affected by vitiligo [1, 4, 5]. On the other hand, repigmentation therapy focuses on restoring pigmentation to the depigmented areas of the skin affected by vitiligo. This can be achieved through various treatment options, including topical corticosteroids, calcineurin inhibitors, phototherapy (such as narrowband UVB or PUVA), and surgical interventions like autologous melanocyte transplantation or micropigmentation [1, 5].

MBEHQ, depigmenting agent used in the treatment of extensive vitiligo, works by inhibiting the enzyme tyrosinase, which is involved in the production of melanin in the skin, leading to the lightening of the skin in areas affected by vitiligo. MBEHQ is typically used in cases of extensive vitiligo where depigmentation of the remaining normal skin can help achieve a more uniform skin tone [4]. Moreover, limited studies have investigated the specific interaction between retinoic acid and MBEHQ regarding melanocyte absorption, and suggested that retinoic acid promotes the absorption of MBEHQ by melanocytes [6, 7]. There is also a hypothesis that needs more study; MBEHQ does possess a quinone functional group, so it is theoretically possible for it to interact with certain quinone receptors to produce neo‐antigens, which are foreign substances that trigger the immune system and kill melanocytes. Van den Boorn et al. showed MBEHQ to increase melanocyte and melanoma cell immunogenicity by forming quinone haptens to the tyrosinase protein and by inducing the release of tyrosinase and melanoma antigen recognized by T cells‐1 (MART‐1)‐containing CD63^+^ exosomes following melanosome oxidative stress induction [8].

Evidence suggest that proinflammatory cytokines such as interleukin‐1 (IL‐1) and interleukin‐18 (IL‐18) may be involved in the pathogenesis of vitiligo [9, 10]. Studies have shown that levels of IL‐1 and IL‐18 may be elevated in the skin of individuals with vitiligo. These cytokines can stimulate the production of other proinflammatory mediators and exacerbate inflammation in the skin [9, 10, 11, 12]. In individuals with vitiligo, an overactive immune response targeting melanocytes leads to their destruction and the development of depigmented patches characteristic of the condition. The exact mechanisms by which IL‐1 and IL‐18 contribute to vitiligo are still being investigated, but it is believed that these cytokines may play a role in triggering and perpetuating the autoimmune response that leads to melanocyte destruction [13]. The aim of present study was to investigate of the effects MEBHQ cream on oxidative stress and its relationship with the aforementioned interleukins in vitiligo patients.

## 2 Materials and Methods

### 2.1 Study Design and Sampling

This interventional and case–control study was conducted on 30 patients (20 men and 10 women between 21 and 50 years old) and 30 healthy people (19 men and 11 women between 20 and 48 years old) referred to different dermatology centers in Iraq. People with vitiligo whose patches covered more than half of their body (50%), who wanted to have a uniform skin color with the patches, or who had no results from repigmentation were included in the study. Due to the irreversible nature of this treatment, the dermatologist had a consultation session with each patient, and after giving them enough time to make a decision, consent was obtained from them to enter the study. Healthy or control people were also selected after confirming their authenticity by a dermatologist. The general exclusion criteria were antioxidant and complementary drug users, pregnant or lactating women, liver and kidney patients, and immunosuppressant drug users.

The Ethics Commission of the Iran University of Medical Sciences (place of designing the project; ethical code: IR.IUMS.REC.1402.1027) and Iraq Ministry of Health (the location of the project; code: 5/5/19651) verified the project's compliance with “Helsinki Laws” and approved the study. After the doctor explained the project and ensured the confidentiality of personal data, interested participants entered with personal and written consent. After confirming the inclusion of each case in the study, blood samples were taken after 12 h of fasting. Then, vitiligo patients were given MBEHQ cream to use on the skin three times a day for 3 months according to the doctor's instructions. The study groups were healthy or control group, vitiligo patients before and after treatment. As monobenzone decreases the amount of melanin in the skin, it makes the skin more sensitive to sunlight and UV light. Therefore, it is crucial for patients using monobenzone cream to protect themselves from excessive sun exposure. So vitiligo patients as well as healthy control group were told to limit time spent in direct sunlight, especially during peak UV radiation hours (usually between 10 a.m. and 4 p.m.), to use a broad‐spectrum sunscreen with a high SPF (sun protection factor) to all exposed areas of the skin before going outdoors and to protect the eyes from UV light by wearing sunglasses that block both UVA and UVB rays. After 3 months of treatment of vitiligo patients with MBEHQ, blood sample was taken. After separating the serum and performing biochemical tests for FBS, HDL, LDL, SGOT, SGPT, BUN, and Cr by related enzymatic kits and autoanalyzer, the separated sera were kept at −70°C until the next tests.

### 2.2 Inflammatory Cytokines

Serum IL‐18 and IL‐1β concentrations were quantified by ELISA kits based on the biotin double‐antibody sandwich technology according to the manufacturer's instructions. Briefly, the serum sample is added to the well coated with the monoclonal IL‐18/IL‐1β antibody and then incubated. Subsequently, anti‐IL‐18/anti‐IL‐1β antibodies labeled with biotin are added, which form a complex with streptavidin‐HRP. Unbound streptavidin is removed after incubation and washing. By adding the substrate, the solution turns blue and turns yellow under the influence of acid (abs. 500 nm). The results were expressed as pg/mL.

### 2.3 Oxidative Stress Profiles

#### 2.3.1 Total Oxidant Status

Total Oxidant Status (TOS) provides information on the total amount of oxidants present in the sample, including reactive oxygen species (ROS) and other free radicals. TOS can be determined with iron (Fe) by Fenton reactions in which Fe^2+^ reacts with free radicals and is oxidized to iron (III). The test was calibrated with H_2_O_2_ (abs. 530 nm). The results were expressed as μmol H_2_O_2_ equiv/mL.

#### 2.3.2 Malondialdehyde

Lipid peroxidation occurs as a result of cell damage in the body. Malondialdehyde (MDA), as a by‐product of lipid peroxidation, is used as a marker to measure the level of oxidative stress in the body and can be measured using the thiobarbituric acid reactive species (TBARS) method. In this method, MDA reacts at high temperatures with TBA to produce the pink‐colored product TBARS, which is measured with a spectrophotometer. MDA assay was carried out based on the conjugation of MDA with 2‐TBA to form a pink product (abs. 535 nm). MDA content was reported as nmol/mL.

### 2.4 Statistics Data Analysis

SPSS (version 22) and Prism software (version 9) were used to analyze the data. The normality of the data was checked using the Shapiro test. The data obtained were expressed as mean ± SD. Unpaired *t*‐tests were used to compare vitilgo patients with the control group and to compare the values of the parameters before and after treatment. *p* < 0.05 was considered statistically significant.

## 3 Result

There was no significant difference between the patient group and the control group in terms of age (33.5 ± 7.2 and 34.1 ± 8.9 respectively, Pv > 0.05). Table 1 compares the vitiligo %, BMI, and biochemical parameters among vitiligo patients and healthy control, before and after MEBHQ therapy, and MEBHQ‐treated pationts with healthy control by unpaired *t*‐tests. MEBHQ therapy significantly increased the area of vitiligo patches (Pv < 0.0001) and made the skin color close to uniformity. The results of the FBS, kidney (BUN and Cr), and liver (ALT and AST) tests showed that vitiligo had no effect on BMI, blood sugar and the function of the pancreas, kidneys, and liver (Pv > 0.05). The results also showed that the use of MEBHQ cream had no side effects on the mentioned parameters and tissues (Pv > 0.05).

**TABLE 1:** Comparison of vitiligo %, BMI, and biochemical factors among the study groups.

Parameters	Groups	Mean ± SD	Sig.
Vitiligo %	(A) Control group	0%	Pv (B vs. C) < 0.0001
	(B) Vitiligo before treatment	62% ± 0.137	
	(C) Vitiligo after treatment	91% ± 0.139	
BMI (kg/m^2^)	(A) Control group	22.53 ± 1.2	Pv (A vs. B) = 0.063
	(B) Vitiligo before treatment	23.36 ± 2.0	Pv (B vs. C) = 0.98
	(C) Vitiligo after treatment	23.37 ± 2.1	Pv (C vs. A) = 0.064
FBS (mg/dL)	(A) Control group	77.3 ± 13.1	Pv (A vs. B) = 0.43
	(B) Vitiligo before treatment	80.1 ± 14.1	Pv (B vs. C) = 0.83
	(C) Vitiligo after treatment	80.9 ± 15.5	Pv (C vs. A) = 0.34
BUN (mg/dL)	(A) Control group	26.2 ± 6.6	Pv (A vs. B) = 0.86
	(B) Vitiligo before treatment	25.9 ± 8.2	Pv (B vs. C) = 0.97
	(C) Vitiligo after treatment	26.0 ± 8.5	Pv (C vs. A) = 0.89
Cr (mg/dL)	(A) Control group	1.18 ± 1.48	Pv (A vs. B) = 0.74
	(B) Vitiligo before treatment	0.90 ± 0.22	Pv (B vs. C) = 0.88
	(C) Vitiligo after treatment	0.89 ± 0.22	Pv (C vs. A) = 0.62
ALT (U/mL)	(A) Control group	22.6 ± 7.9	Pv (A vs. B) = 0.07
	(B) Vitiligo before treatment	19.2 ± 6.3	Pv (B vs. C) = 0.96
	(C) Vitiligo after treatment	19.2 ± 6.4	Pv (C vs. A) = 0.07
AST (U/mL)	(A) Control group	24.7 ± 5.4	Pv (A vs. B) = 0.50
	(B) Vitiligo before treatment	25.8 ± 6.8	Pv (B vs. C) = 0.86
	(C) Vitiligo after treatment	26.1 ± 7.1	Pv (C vs. A) = 0.40

### 3.1 Effects of Treatment on Inflammatory Cytokines

**FIGURE 1:**
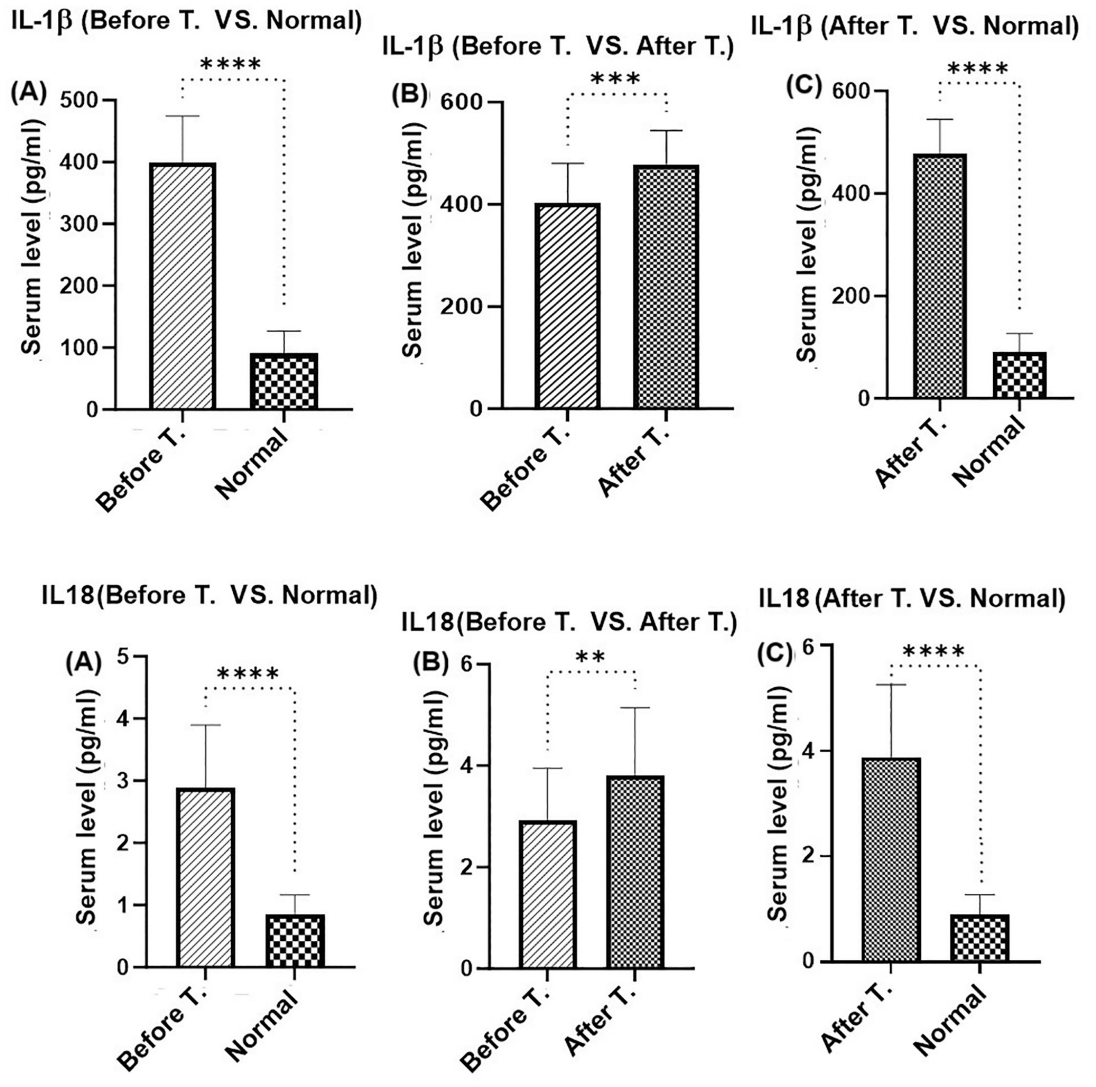
Comparision of IL‐1B and IL‐18 levels in vitiligo patients "A and D, respectively: before treatment versus normal group", "B and E, respectively: before treatment versus after treatment", "C and F, respectively: after treatment versus normal group. **p < 0.01; ***p < 0.001; ****p < 0.0001.

Figure 1 compares the total blood levels of IL‐1β (Figure 1A) and IL‐8 (Figure 1B) levels of vitiligo patients with healthy control, then compares the levels of mentioned factors before and after using MEBHQ. Unpaired *t*‐test analysis showed significantly higher blood levels of IL‐1β and IL‐8 in vitiligo patients compared to healthy subjects (Figure 1A,D, Pv < 0.0001). MEBHQ‐treatment further increased the levels of these inflammatory factors in vitiligo patients, compared to before treatment (Figure 1B,E, Pv < 0.01) and normal control (Figure 1C,F, Pv < 0.0001).

## 4 Effects of Treatment on Oxidative Stress and Biomarker

**FIGURE 2:**
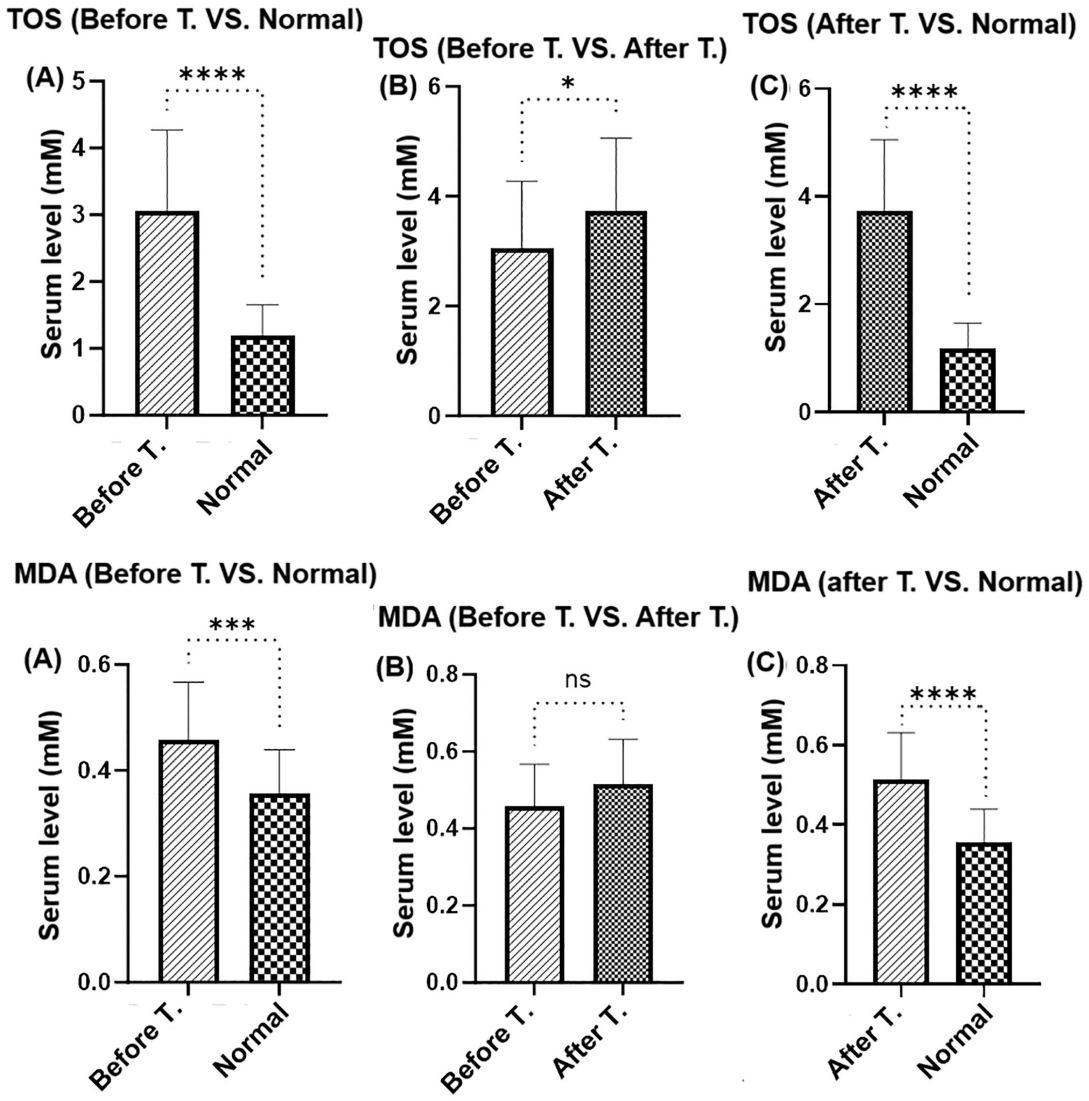
(A) Comparison of TOS and (B) MDA levels in vitiligo patients before and after treatment with MEBHQ cream and in the normal control. *p < 0.1; **p < 0.01; ***p < 0.001; ****p < 0.0001.

Figure 2 compares the total blood levels of TOS (Figure 2A) and MDA (Figure 2B) levels of vitiligo patients with healthy control, then compares the levels of mentioned factors before and after using MEBHQ. Unpaired *t*‐test analysis showed significantly higher blood levels of TOS and MDA in vitiligo patients compared to healthy subjects (Figure 1A,D) (Pv < 0.0001). MEBHQ treatment increased TOS and MDA levels in vitiligo patients, but TOS increase was statistically significant (Figure 2b, Pv < 0.0001) and MDA increase was not statistically significant (Figure 2e, Pv > 0.05), and still the level was significantly higher than normal control (Figure 2c,f, Pv < 0.0001).

### 4.1 Relation Between Percentage of Vitiligo % With IL‐1β, IL‐18, TOS, and MDA

Spearman correlation between percentage of vitiligo % with metabolic parameters of IL‐1β and IL‐18 was determined. There were positive correlations among percentage of vitiligo (%) and the levels of both IL‐1β and IL‐18 as well as both interleukins together as shown in Table 2A. Regression analysis of IL‐1β and IL‐18 on percentage of vitiligo showed a positive association with IL‐1β (*B* = 0.812, *p* = 0.001) and IL‐18 (*B* = 0.149, *p* = 0.018).

**TABLE 2:** Relation between percentage of vitiligo (%) with (A) IL‐1β and IL‐18, (B) TOS, and MDA.

(A) Correlations			
	Vitiligo %	IL‐1beta (pg/L)	IL‐18 (ng/L)
Vitiligo %	1.000		
IL‐1β	0.858**	1.000	
IL‐18	0.794**	0.801**	1.000

(B) Correlations			
	Vitiligo %	MDA (nM)	TOS (mM)
Vitiligo %	1.000		
MDA	0.662**	1.000	
TOS	0.811**	0.816**	1.000

**Correlation is significant.

Spearman correlation between percentage of vitiligo % with metabolic parameters of TOS and MDA was determined. There are positive correlations among percentage of vitiligo (%) and the levels of both TOS and MDA as well as both parameters shows in Table 2B. Regression analysis of TOS and MDA on percentage of vitiligo showed a positive association with TOS (*B* = 0.871, *p* = 0.0001) and no significant association with MDA (*B* = −0.094, *p* = 0.432). In this study, as the TOS variable has a larger standardized coefficient (*B* = 0.898), TOS played a more effective role in predicting vitiligo disease status than MDA.

### 4.2 Receiver Operating Characteristic Curves

#### 4.2.1 Validity of IL‐1β and IL‐18 and TOS and MDA Tests Among Vitiligo Patients Before Treatment and Normal Control

**FIGURE 3:**
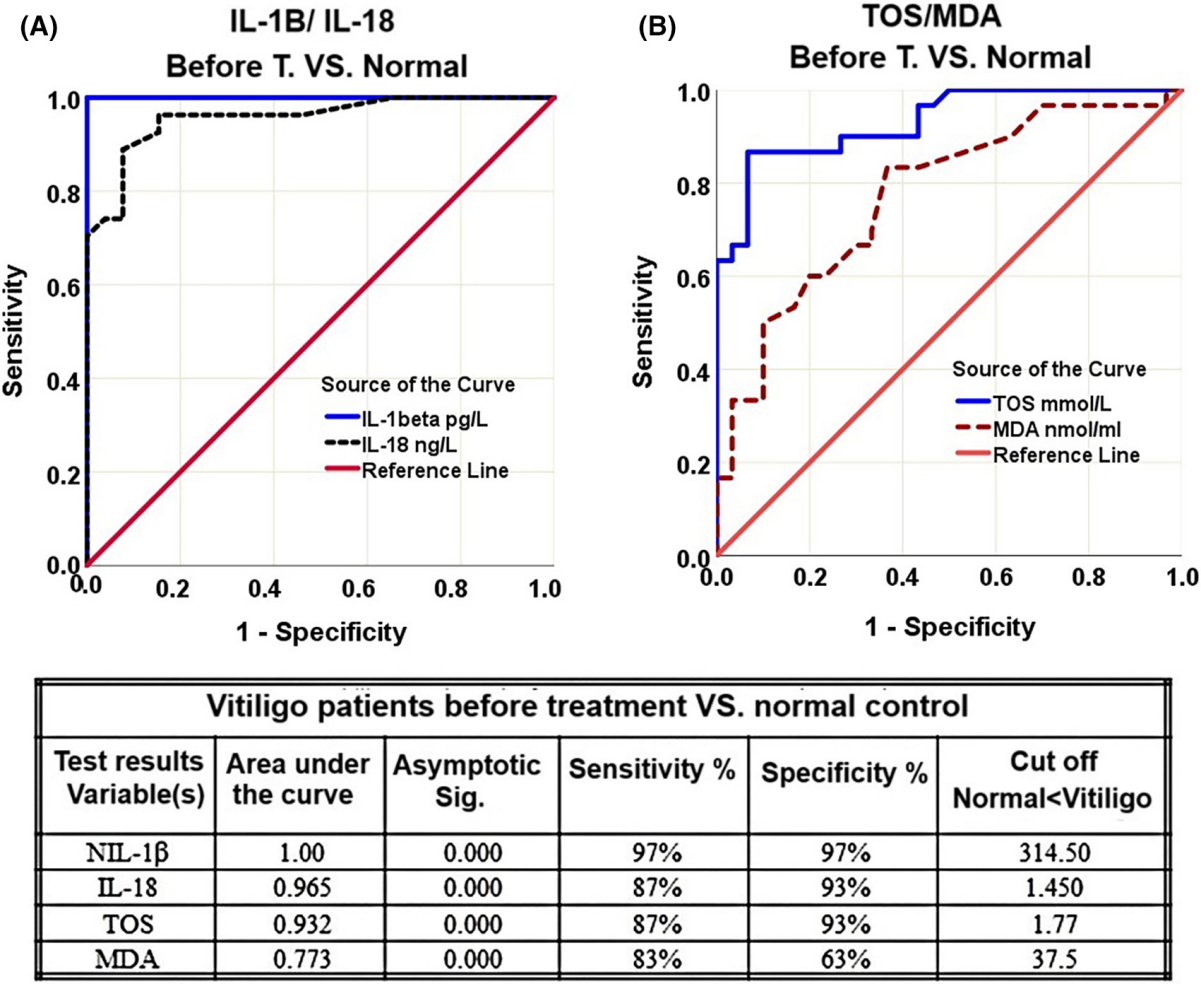
ROC curve for IL‐1β and IL‐18 (A), and TOS and MDA (B) among vitiligo patients before treatment and normal control. Table shows ROC analysis of the mentioned parameters.

Receiver operating characteristic (ROC) curve was used to evaluate the validity tests of IL‐1β and IL‐18 cytokines (Figure 3A) as well as oxidative biomarkers of TOS and MDA (Figure 3B) in vitiligo patients before treatment versus healthy group. Both curves are skewed from the 45° diagonal. The cutoffs with the best equilibrium between sensitivity and specificity of the mentioned factors have been brought in the table included in Figure 3.

##### 4.2.1.1 Cytokines

The curve for IL‐1β is more shifted up and to the left than IL‐18. The AUC (area under the curve) of both factors are all significantly higher than 0.5 (*p* < 0.05). The sensitivity and specificity of IL‐1β is higher than IL‐18.

##### 4.2.1.2 Oxidative Factors

The curve for TOS is more shifted up and to the left than MDA. The AUC of both factors were all significantly higher than 0.5 (*p* < 0.05). The sensitivity and specificity of TOS is higher than MDA.

#### 4.2.2 Validity of IL‐1β and IL‐18, and TOS and MDA Tests After Using MEBHQ

**FIGURE 4:**
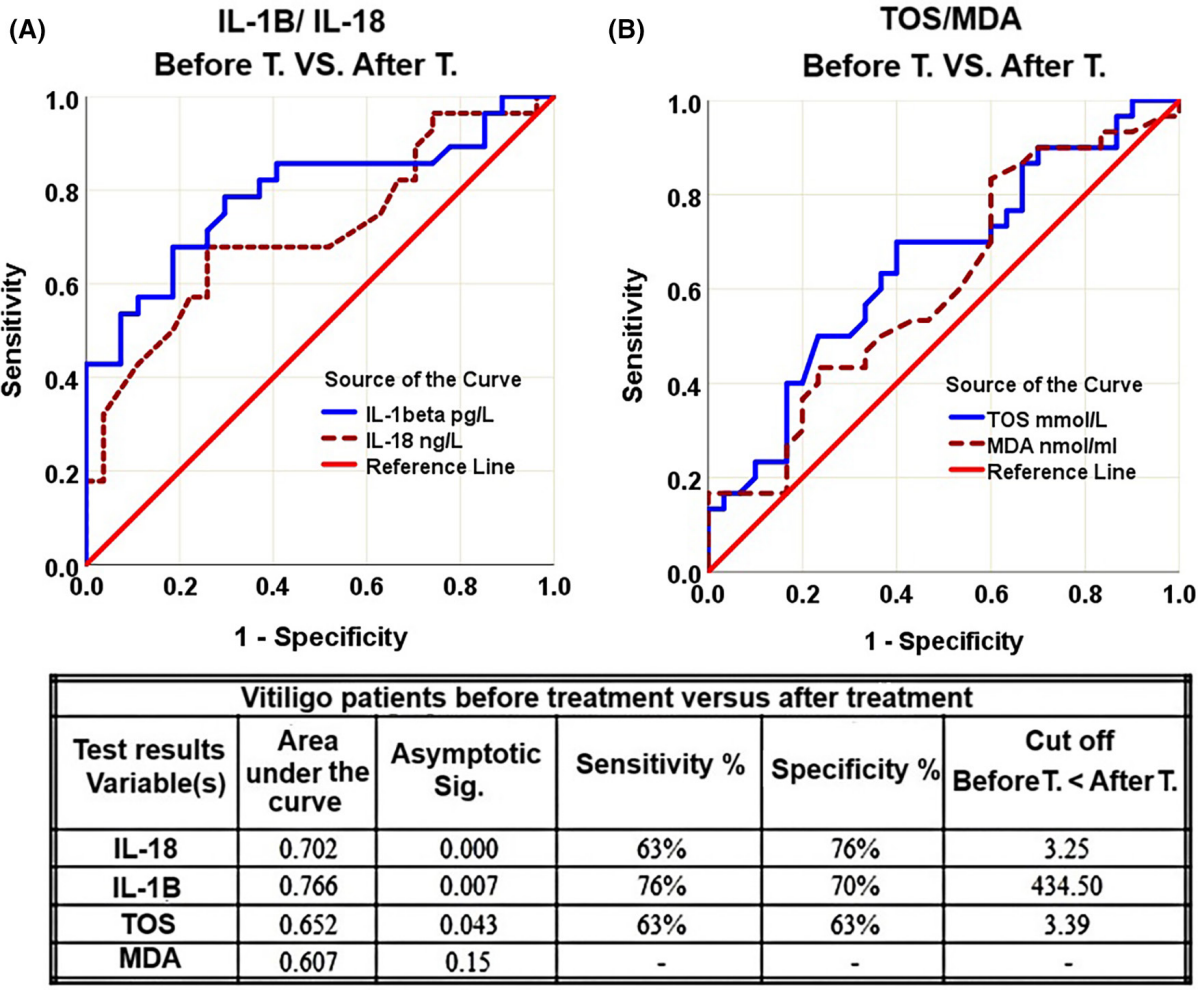
ROC curve for IL‐1β and IL‐18 (A), and TOS and MDA (B) among vitiligo patients before and after treatment with MBEHQ. Table shows ROC analysis of the mentioned parameters.

ROC curve was used to evaluate the response to MEBHQ based on changes in blood levels of IL‐1β and IL‐18 cytokines (Figure 4A) as well as oxidative biomarkers of TOS and MDA (Figure 4B). Both curves are skewed from the 45° diagonal. The cutoffs with the best equilibrium between sensitivity and specificity of the mentioned factors, have been brought in the table included in Figure 4.

##### 4.2.2.1 Cytokines

The curve for IL‐1β is more shifted up and to the left than IL‐18. The AUC of both factors are all significantly higher than 0.5 (*p* < 0.05). The sensitivity of IL‐1β is higher than IL‐18 but the specificity of IL‐18 is partially higher than IL‐1β.

##### 4.2.2.2 Oxidative Factors

The curve for TOS is more shifted up and to the left than MDA. The AUC of TOS was significantly larger than 0.5 (*p* < 0.05) but it is not significant for MDA. The ROC curve for MDA is not valid.

## 5 Discussion

Vitiligo patients may choose depigmentation treatments for several reasons, despite the desire for pigmentation [14]. MBEHQ, a more potent and modified hydroquinone, disrupts the melanin production process at the enzymatic level, leading to a reduction in melanin pigment in the skin and eventual depigmentation in targeted areas [15]. Our results also showed a 60%–90% decrease in the skin patches of vitiligo patients after 3 months of using crème. Limited research exists on the long‐term use of monobenzone cream for vitiligo treatment, and specific studies on the side effects of prolonged use are scarce. However, potential side effects of extended monobenzone cream use may include hypopigmentation, skin irritation, increased sensitivity to sunlight, psychological impacts related to changes in skin pigmentation, and most importantly, oxidative stress.

Previous research has shown that vitiligo patients often exhibit higher levels of oxidative stress markers including ROS, lipid peroxidation products, and decreased antioxidant defenses compared to healthy individuals [16, 17]. Our results also showed higher levels of oxidative stress and lipid peroxidation products in vitiligo patients compared with healthy people. Because, these patients often exhibit lower levels of antioxidants such as catalase, superoxide dismutase, and glutathione. Oxidative stress can lead to DNA damage, which may contribute to the destruction of melanocytes in vitiligo patients [18]. Comparably, individuals with non‐segmental vitiligo were the subjects of a cross‐sectional, analytical investigation by De Sarkar et al. The findings showed that patients with active vitiligo had increased levels of ROS, high levels of MDA, AND IL‐6, TNF‐a, IL‐1b, IFN, and IL‐8. Together, these studies findings have demonstrated that increased ROS production in vitiligo results in oxidative damage, especially to lipids and DNA, and is associated with a proinflammatory cytokine milieu [19]. According to Xuan et al.'s study on oxidative stress, they also showed that disturbance of the balance between oxidants and antioxidants as a result of increased levels of free oxygen radicals resulting from various conditions has a fundamental role in the disorders that occur in melanocytes and leads to the formation of vitiligo disease [18].

Generally, hydroquinone increases oxidative stress through a process known as auto‐oxidation, leading to the formation of reactive intermediates and free radicals. These reactive species can then interact with cellular components, such as lipids, proteins, and DNA, causing oxidative damage [20]. Additionally, MBEHQ inhibits the activity of the enzyme catalase, which leads to an accumulation of hydrogen peroxide in the skin, further contributing to oxidative stress [21]. As our results also showed, the increase in oxidative stress induced by MBEHQ plays a role in the depigmentation process by damaging melanocytes and reducing their ability to produce melanin pigment. However, in the current study, following 3 months of treatment with MBEHQ and the positive outcome of depigmentation, there was no significant increase in the damage index, specifically lipid peroxidation, despite the elevated levels of total TOS observed compared to pretreatment levels. It seems that 3 months of treatment is a suitable period for treatment and prevention of side effects.

There is a complex interplay between oxidative stress and the immune system in vitiligo, creating a feedback loop that perpetuates disease progression [18]. ROS can activate immune cells, such as dendritic cells and T lymphocytes, which in turn produce more ROS and inflammatory mediators, leading to further stimulation of the autoimmune response against melanocytes and contributing to the progression of vitiligo [22]. As our study showed high levels of serum IL‐1β and IL‐18 in vitiligo patients, activated dendritic cells in this disease can produce IL‐1β and IL‐18 in response to inflammatory stimuli. These cytokines can activate dendritic cells in a positive loop, promoting their maturation and antigen‐presenting capabilities. Activated dendritic cells can then present melanocyte‐specific antigens to autoreactive T lymphocytes, leading to their activation and differentiation into effector T cells targeting melanocytes [23]. T lymphocytes, particularly CD8^+^ cytotoxic T cells, are key effectors in the autoimmune destruction of melanocytes in vitiligo. Additionally, IL‐1β and IL‐18 can stimulate the activation and proliferation of autoreactive T cells, promoting their differentiation into cytotoxic T cells that specifically target melanocytes. These activated T cells produce pro‐inflammatory cytokines such as IFN‐γ and TNF‐α, which further drive inflammation and melanocyte damage in vitiligo [24]. Moreover, IL‐1β and IL‐18 can directly induce apoptosis (cell death) in melanocytes or disrupt their function, leading to depigmentation in vitiligo [25], which increased after using MBEHQ by vitiligo patients of this study. The specific interactions between MBEHQ and IL‐1β and IL‐18 have not been extensively studied and there is limited information on direct interactions between hydroquinone and these cytokines. However, our study showed increased levels of oxidative stress as well as IL‐1β and IL‐18 in vitiligo patients after using MBEHQ cream. As IL‐1β and IL‐18 are pro‐inflammatory cytokines in the autoimmune response in vitiligo, their increased blood levels after MBEHQ cream application may be due to increased ROS. However, as MBEHQ induces melanocyte destruction and alters the skin microenvironment, it may influence the production or activity of these cytokines in the skin. On the other hand, since MBEHQ induces melanocyte destruction and alters the skin microenvironment, it may influence the production or activity of these cytokines in the skin [26].

High levels of oxidative stress and inflammation can have potential adverse effects on various organs, including the liver, pancreas, and kidneys [27]. However our results did not show abnormal changes in the liver, kidney, and pancreas after 3 months of MBEHQ cream consumption; it is possible that the 3‐month consumption period considered in this study was not long enough for such side effects to occur in the body. Possibly, over time, chronic exposure to MBEHQ‐induced oxidative stress contribute to liver inflammation, fibrosis, and even liver damage [28]. Chronic exposure of the pancreas to MBEHQ‐induced oxidative stress may disrupt pancreatic function, impair insulin production, and contribute to the development of pancreatic disorders such as pancreatitis or diabetes [29]. Chronic exposure of the kidney to MBEHQ‐induced oxidative stress may contribute to the development of kidney disorders such as nephritis, renal fibrosis, or even kidney failure [30]. In summary, MBEHQ‐induced oxidative stress can have detrimental effects on the liver, pancreas, and kidneys by causing cellular damage, inflammation, and dysfunction in these organs [31]. Therefore, it is important to monitor for signs of organ damage while using MBEHQ and to consult with a healthcare provider if any concerning symptoms arise. The use of antioxidants and other protective actions during treatment with MBEHQ is a very important issue that should be addressed in future research. However, the 60%–90% reduction in vitiligo patches shown in this study could promise short‐term use and the absence of side effects in these people.

## 6 Conclusions

In vitiligo, oxidative stress plays a significant role in triggering and exacerbating the autoimmune response against melanocytes. Overall, while the direct relationship between hydroquinone, MBEHQ, oxidative stress as well as IL‐1β, and IL‐18 with vitiligo is not well‐established, it is possible that these depigmenting agents could indirectly more stimulate the immune responses. However, the presence of these cytokines in the skin microenvironment contributes to the autoimmune attack on melanocytes and MBEHQ intensifies this process to more damage and inhibit melanin production by melanocytes. Although, the 60%–90% increase in vitiligo patches shown in this study could promise short‐term use and the negligible side effects in these people, chronic exposure of body to MBEHQ‐induced oxidative stress may contribute to the development systemic disorders including liver, kidney, pancreas, and heart failures. Therefore, according to the principles of drug therapy monitoring (TDM), it is suggested to measure the effective dose of the drug and even its blood absorption in people in subsequent studies in order to minimize oxidative stress and side effects of MBEHQ cream.

## Conflicts of Interest

The authors declare no conflicts of interest.

## Data Availability

The data that support the findings of this study are available on request from the corresponding author. The data are not publicly available due to privacy or ethical restrictions.
